# Transition from fractional to classical Stokes–Einstein behaviour in simple fluids

**DOI:** 10.1098/rsos.170507

**Published:** 2017-12-13

**Authors:** Diego Coglitore, Stuart P. Edwardson, Peter Macko, Eann A. Patterson, Maurice Whelan

**Affiliations:** 1 Institut Européen des Membranes, Montpellier, France; 2 School of Engineering, University of Liverpool, Liverpool, UK; 3 European Commission, Joint Research Centre (JRC), Ispra, Italy

**Keywords:** diffusion, Stokes–Einstein diffusion, fractional Stokes–Einstein equation, single nanoparticle tracking, nanoparticles

## Abstract

An optical technique for tracking single particles has been used to evaluate the particle diameter at which diffusion transitions from molecular behaviour described by the fractional Stokes–Einstein relationship to particle behaviour described by the classical Stokes–Einstein relationship. The results confirm a prior prediction from molecular dynamic simulations that there is a particle size at which transition occurs and show it is inversely dependent on concentration and viscosity but independent of particle density. For concentrations in the range 5 × 10^−3^ to 5 × 10^−6^ mg ml^−1^ and viscosities from 0.8 to 150 mPa s, the transition was found to occur in the diameter range 150–300 nm.

## 1. Introduction

It is well known that the motion of molecules and particles in a fluid is different. In molecular diffusion, the diffusing species is similar or identical in size to the solvent, whereas particles are larger than the solvent species. The diffusion of particles follows the Stokes–Einstein relationship while the diffusion of fluid molecules, such as water, does not satisfy the assumptions underlying the Stokes–Einstein relationship and is better described by a fractional relationship [1,2]. The issue investigated in this study was the conditions at which diffusion behaviour transitions from one type of behaviour to the other; in other words, how large a cluster of molecules, in the form of a solute particle, is required to induce Stokes–Einstein-type motion in a simple fluid?

Others have considered the change in diffusive behaviour with particle size in dense fluids, for example Rudyak *et al.* [3], and Ould-Kaddour & Levesque [4], and at extreme temperatures [5,6]. However, relatively little attention has been paid to diffusion at close to room temperature in simple fluids. Molecular dynamics simulations have been used to establish that, in a simple fluid, a hydrodynamic regime is replaced by a Stokes–Einstein regime when the mass or size ratio of the solute to solvent molecules is raised above approximately four for an infinite dilution of solute in a solvent [7]. Subsequently, molecular dynamics simulations were used to establish that a fractional Stokes–Einstein relationship could be used to represent diffusion at the molecular scale [8,9], including for the self-diffusion of water [10] over temperatures from 238 K to 363 K [1]. More recently, a fractional Stokes–Einstein equation was used to fit new experimental data for water diffusion in the same temperature range [11]. In this context, it should be noted that the transport and thermodynamic behaviour of water render it an almost unique substance.

Li [12] reviewed several studies and concluded that there is a critical size of particle below which a hydrodynamic regime dominates with van der Waals forces between the solvent molecules and solute molecule or particle playing a fundamental role; and above which particle diffusion can be described by the Stokes–Einstein relationship. However, the challenges involved in obtaining data directly from the diffusion of individual particles has obstructed the confirmation by experiment of the critical size because the velocities render scanning instruments impractical and because the critical size must lie below the diffraction limit for optical microscopes. Support for the latter is provided by the experiments of Lurio *et al.* [13] and Koenig *et al.* [14] who have demonstrated the validity of the Stokes–Einstein relationship at diameters of the order of 150 nm for polystyrene particles in glycerol using X-ray photon correlation spectroscopy and for gold nanoparticles in water using dark-field microscopy.

This study was conducted in two parts. First, a commercially available dynamic light scattering (DLS) system was used to identify the approximate limit of the Stokes–Einstein relationship. In the second part, a recently developed optical tracking technique based on caustics [15] was used to track individual particles over a range of sizes, concentrations and viscosities in order to better define the transition between the fractional and classical Stokes–Einstein behaviour. The tracking method based on caustics involves no assumptions about the nature of the motion of the particles and instead allows the position of a particle to be identified directly. However, the dynamic light scattering methods use the classical Stokes–Einstein relationship in the analysis of measurement data; and hence would not be expected to provide reliable results for particles exhibiting a behaviour better described by a fractional Stokes–Einstein relationship. Some previous investigations have approached the limit of reliable results; for example, Sabuncu *et al.* [16] sized gold nanoparticles of nominal diameters 10, 25, 50 and 100 nm at a concentration of 5 × 10^−3^ mg ml^−1^ in water but measured average diameters of 24, 41, 65 and 97 nm with substantially larger scatter in the results at the smaller diameters; Egerton & Tooley [17] reached a similar conclusion for TiO_2_ particles between 7 and 200 nm diameter at a concentration of 0.1% by weight; while Lee *et al.* [18] found that DLS was unable to identify 50 nm silica particles in a mixture of 50 nm and 100 nm diameter particles. These results confirm that both size and concentration are important in defining the transition from fractional to classical Stokes–Einstein behaviour.

Hence, exploring the limit of reliability of a dynamic light scattering system allows the transition from fractional to classical Stokes–Einstein behaviour to be identified relatively easily though imprecisely. The optical tracking technique was used to confirm and define the transition more precisely, though these experiments required considerable additional effort.

**Figure 1.**
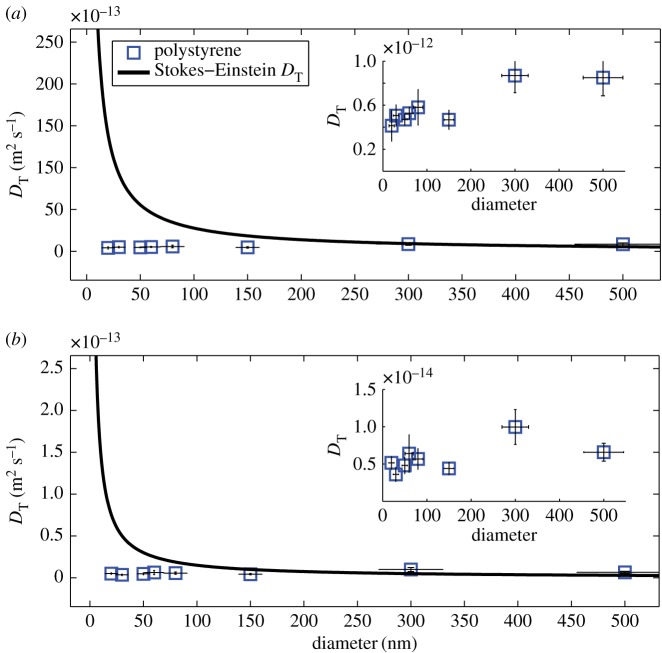
Diffusion coefficient, *D*_T_ for polystyrene particles of diameter from 10 to 500 nm calculated from measured mean square displacements and predicted using the Stokes–Einstein relation at a concentration of 5 × 10^−6^ mg ml^−1^ in (*a*) water of viscosity 0.8 mPa s and (*b*) a 9 : 1 glycerol–water mixture of viscosity 150 mPa s.

**Figure 2.**
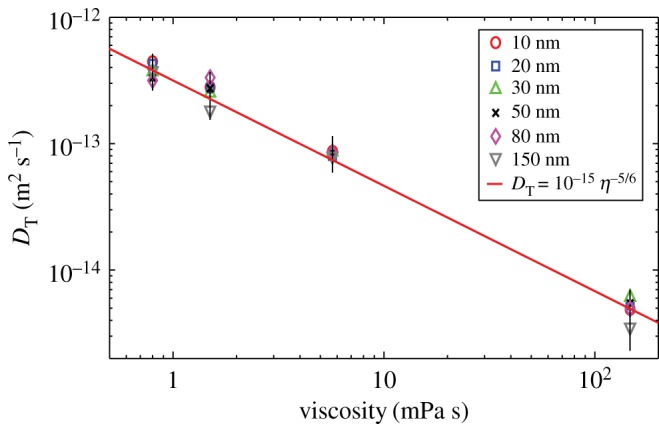
Diffusion coefficient, *D*_T_ for gold particles ranging in diameter from 10 to 150 nm diameter calculated from measured mean square displacements as a function of viscosity together with the fractional Stokes–Einstein equation fitted to the data.

**Figure 3.**
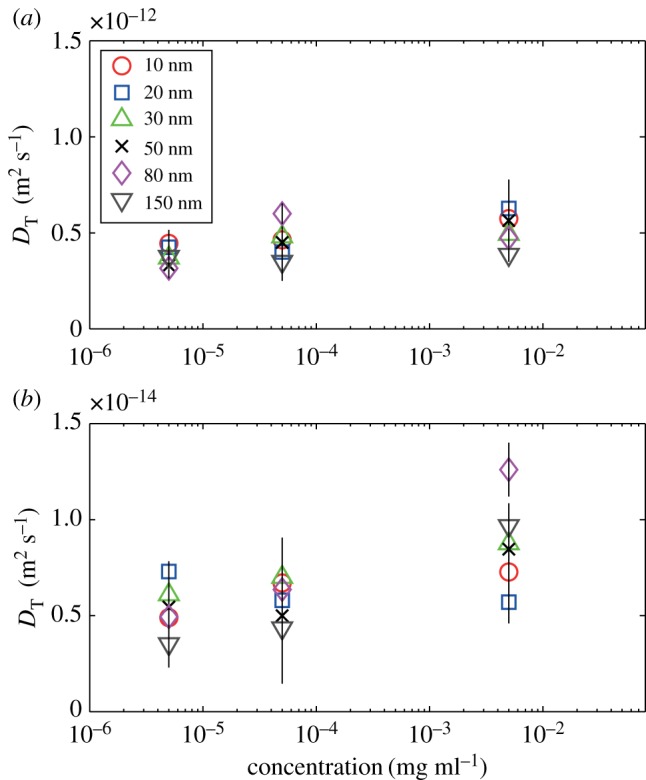
Diffusion coefficient, *D*_T_ for gold particles, ranging in size from 10 nm to 150 nm, calculated from measured mean square displacements as a function of concentration in (*a*) water with a viscosity of 0.8 mPa s and (*b*) a 9 : 1 glycerol–water mixture with a viscosity of 150 mPa s.

**Figure 4.**
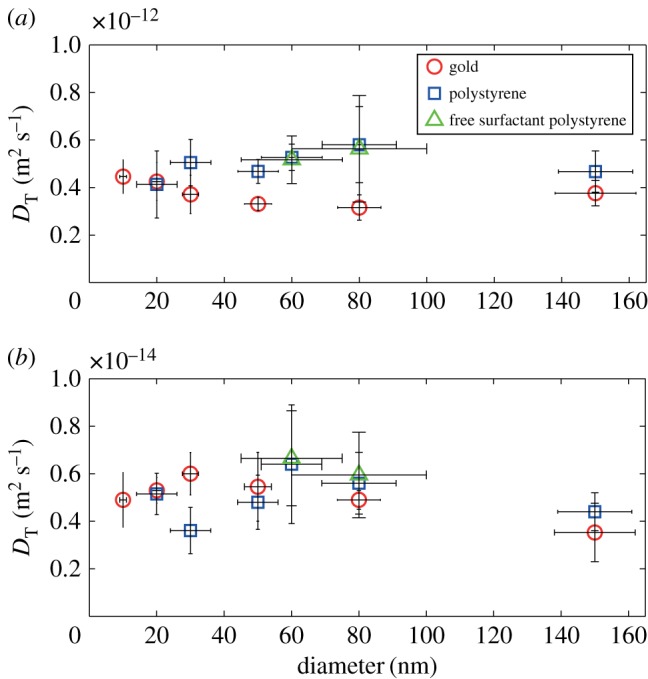
Diffusion coefficient, *D*_T_ for gold (nominal density of 19.3 g cm^−3^) and polystyrene (nominal density of 1.05 g cm^−3^) particles as a function of particle diameter in (*a*) water with a viscosity of 0.8 mPa s and (*b*) a 9 : 1 glycerol–water mixture with a viscosity of 150 mPa s.

We used a Nanosight system (Nanosight LM10, Malvern Instruments Ltd) and explored its reliability in identifying the size of gold particles of diameter 20, 80 and 150 nm. The latter diameter corresponds to the level at which Lurio *et al.* [13] and Koenig *et al.* [14] have confirmed Stokes–Einstein behaviour, while the smaller diameters are dimensions at which others have encountered unreliable measurements. We found the 150 nm particles could be correctly sized at a concentration of 5 × 10^−2^ mg ml^−1^ but at lower concentrations (5 × 10^−3^ and 5 × 10^−6^ mg ml^−1^) the size was found to be approximately 225 nm (table 1); at these concentrations both the 20 and 80 nm diameter particles were sized at around 200 nm. Using these results as an initial estimate of the transition from fractional to classical Stokes–Einstein behaviour, we have tracked gold and polystyrene particles ranging in size from 10 nm to 500 nm diameters at concentrations ranging from 5 × 10^−3^ to 5 × 10^−6^ mg ml^−1^ in liquids ranging in viscosity from 150 to 0.8 mPa s. The results in figures 1–4 demonstrate that below 150 nm diameter the diffusion of the particles is well described by the fractional Stokes–Einstein relationship, while above approximately 150 nm diameter classical Stokes–Einstein starts to describe the diffusion, although the transition appears to be at a larger diameter with lower viscosities and concentrations.

**Table 1.** Particle size measured using Nanosight system.

	nominal diameter		
concentration (mg ml^−1^)	20 nm	80 nm	150 nm
5 × 10^−6^	202 ± 30 nm	190 ± 28 nm	215 ± 22 nm
5 × 10^−3^	220 ± 34 nm	202 ± 15 nm	236 ± 20 nm
5 × 10^−2^			153 ± 18 nm

## 2. Material and methods

We purchased commercial, spherical, gold (BBI Solutions OEM Limited) and polystyrene (Magsphere Inc.) particles suspended in water with nominal diameters from 500 nm down to 10 nm. It was not possible to obtain spherical gold particles with diameters of 300 nm and 500 nm and so experiments were only performed using polystyrene at these large diameters. Since all these particles were stabilized with surfactants, which might influence their behaviour, we also tested some surfactant-free polystyrene particles (Magsphere Inc.), to look for possible effects of the stabilizing agent on the particle dynamics. We investigated the effect of viscosity on the motion of the particles by using four concentrations of glycerol in distilled water, i.e. volume fractions of 0%, 20%, 50% and 90%, which yielded values of viscosity from 0.8 mPa s for distilled water to 150 mPa s for a 9 : 1 mixture of glycerol and water, i.e. a range of three orders of magnitude to allow the effect of viscosity to be reliably quantified. The value of viscosity was evaluated for each solution using the empirical formula proposed by Cheng [19] and, for one solution (9 : 1 mixture of glycerol and water) it was confirmed, by making measurements using a rheometer (AR1000N, TA Instruments), that adding the particles to the solution had no significant effect (at a 95% confidence level) on the viscosity. The particles were supplied in vials at very high concentrations that varied from 10^9^ particles ml^−1^ for the 150 nm diameter particles to 10^12^ particles ml^−1^ for the 10 nm diameter particles. The concentrations were reduced substantially by adding the concentrate to water or water–glycerol mixtures as appropriate to obtain concentrations of 5 × 10^−3^, 5 × 10^−5^ and 5 × 10^−6^ mg ml^−1^, which corresponded to 10^11^, 10^9^ and 10^8^ particles ml^−1^ respectively for the 10 nm diameter particles. A mixer (Vortex-Genie 2 G560E, Scientific Industries Inc.), together with an ultrasonic bath (U50, Ultrawave), were employed to obtain a uniform particle distribution and avoid aggregation. We confirmed the size and shape of the particles by air-drying them on copper grids for 5 min and then viewing them in a transmission electron microscope (Tecnai G2, Fei) at 120 kV and typical images are shown in electronic supplementary material, figure S1. The resultant solutions were placed in glass microscope slides that had a central circular cavity of depth 0.8 mm and radius 7.5 mm, which then was covered by a 0.16 mm coverslip. The measurements were made in a standard inverted optical microscope (Axio Observer.Z1 m, Carl Zeiss) which was mounted on anti-vibration feet (VIBe, Newport) to isolate the sample from the environment. To isolate the sample from variations in ambient temperature, the solution was maintained at a constant *T* = 303 K using a heated microscope stage (Heatable universal mounting frame KH S1, Pecon GmbH) connected to a controller (Tempcontroller 2000-2, Pecon GmbH) and monitored, for further confirmation of the temperature value, by two thermocouples.

The particles were below the diffraction limit for visible light and hence were not ordinarily visible in the microscope. Some simple adjustments were made to the microscope following the procedure described by Patterson & Whelan [15]. The light source (100 W halogen lamp) and condenser lens were set up for Kohler illumination. Then a narrowband filter (550 ± 2 nm) was inserted and the condenser aperture was closed to leave only a pin-hole through which light was transmitted. When the microscope is adjusted out of focus, these optical conditions cause a caustic [20] to be formed by each particle which is several orders of magnitude larger than the particle and can be used to identify the location of the particle. Caustics have been used in engineering science to evaluate the deformation at geometric discontinuities associated with contact and fracture [21]. They were first explained by Hamilton in 1828 [20] who observed light rays being reflected from curved mirrors, such that light rays were concentrated in some domains and absent from others, and when projected on a flat screen corresponding bright curves and dark areas were observed. Subsequently, the formation of caustics by light transmission through natural objects, such as a rain drop, was explained by Lock & Andrew [22], who formally defined a caustic as the envelope of light rays reflected or refracted by a curved surface or object, or the projection of that envelope of rays onto another surface.

Tracking was performed using a ×40 microscope objective and recording videos with a monochrome CCD camera (AxioCam ICm 1, Carl Zeiss) at 28 fps. The videos were processed using specially written software based on feature-matching routines (LabVIEW NI IMAQ Vision, National Instruments) and the earlier work of Neal *et al.* [23] and allowed the frame-by-frame in-plane co-ordinates of an individual particle to be obtained from the image of the caustic. When the particles aggregate the caustic changes shape from a simple circular shape (electronic supplementary material, figure S2) to a set of overlapping circular shapes (electronic supplementary material, figure S3) so that it was straightforward to ensure single particles were tracked.

The motion of particles can be characterized by their mean square displacement, MSD [24] such that

2.1 $$\text{MSD}=\frac{1}{N-n}\overset{N-n}{\underset{i=0}{\sum }}({\left(x_{i+n}-x_{i}\right)}^{2}+{\left(y_{i+n}-y_{i}\right)}^{2}),$$

where (*x_i_*,*y_i_*) are the coordinates of a particle at the *i*th step; and the diffusion coefficient, *D* is defined as

2.2 $$\text{MSD}(t)=4D_{T}t,$$

in two-dimensional space. For classical Stokes–Einstein behaviour [25],

2.3 $$D_{T}=\frac{k_{b}T}{3\pi \eta d},$$

where *k*_b_ is the Boltzmann constant, *T* is temperature, *η* is fluid viscosity and *d* the diameter of the particle. We calculated the mean diffusion coefficient *D*_T_, over a period of approximately 10 s from the squared displacement for a time interval, *t* following the approach of Ernst & Köhler [26]. The time interval *t*, in equation (2.2), is the time lapse between measurements and is constrained by the frame rate of the camera, which was 28 fps. Michalet has discussed that, in theory, a higher frame rate could improve the accuracy of measurements [27], but in practice we found no significant differences between diffusion coefficients calculated at 28 fps and 500 fps, e.g. *D*_T_ = (0.413 ± 0.16) × 10^−12^ m^2^ s^−1^ and *D*_T_ = (0.426 ± 0.01) × 10^−12^ m^2^ s^−1^ respectively for 20 nm diameter polystyrene particles in water. At the lower frame rate, the caustics were better defined which supported a more robust analysis, while at the higher frame rate the illumination level was low enough to cause some loss of details.

## 3. Results

The values for the diffusion coefficient, *D*_T_ are shown in figure 1 based on equation (2.2) using measurements for the full range of particle sizes that were tracked, i.e. from 10 to 500 nm diameters, together with the corresponding prediction using the classical Stokes–Einstein relationship in equation (2.3). It can be seen that the transition to classical Stokes–Einstein behaviour occurs for particles with a diameter of about 300 nm at a viscosity of 0.8 mPa s, but with a diameter of about 150 nm at a viscosity of 150 mPa s, when the prediction lies on the edge of the error bar for the measurements. The validity of the Stokes–Einstein relationship above this size scale has been demonstrated in a number of studies including by Li *et al.* [28] and by Huang *et al.* [29]; so, no further measurements were made for larger diameter particles. In figure 2, the measured diffusion data for particles with a diameter below the transition, i.e. ≤150 nm, have been plotted as a function of viscosity to demonstrate the dependency is consistent with fractional Stokes–Einstein behaviour. The term ‘fractional’ refers to the diffusion coefficient, *D*_T_ being inversely proportional to a fractional power of viscosity, such that

3.1 $$D_{T}=\frac{C}{\eta^{p}},$$

where *C* and *p* are constants and *p* is less than unity [10], for example 0.79 < *p* < 1 for molecular and ionic liquids [1]. For all of the data obtained below the transition or critical value, i.e. *d* ≤ 150 nm, the two parameters, *C* and *p*, were found to be independent of the size and density of the particles with values of *p* = 0.84 and *C* = 10^−15^ for all concentrations tested with regression coefficients of *R*^2^ = 0.98 at 5 × 10^−6^ and 5 × 10^−5^ mg ml^−1^, and *R*^2^ = 0.96 at 5 × 10^−3^ mg ml^−1^. The slightly poorer regression coefficient is a result of a greater scatter in the diffusion coefficient, *D*_T_ at the higher concentration particularly for gold particles in the high-viscosity solution, as shown by the data in figure 3. It is apparent that the transition from fractional to classical Stokes–Einstein behaviour begins at a smaller diameter with higher concentration and viscosity as indicated in figure 3*b*.

A direct comparison of the behaviour of gold and polystyrene particles below the transition size is shown in figure 4 together with results for surfactant-free particles. The latter demonstrate that the surfactant has no influence on the measurements. Some surfactant is used in the production of the surfactant-free particles so that there may be trace amounts present but these are expected to have a negligible effect. Error bars are included in figure 4 based on data from the manufacturer about the uncertainty on the size of the particles for the horizontal direction; and in vertical direction, on the standard deviation of six measurements of the mean square displacement for each material and size. There were no significant outliers in the data, which might have been expected if any mis-sized particles were present in the sample; although examination of the particles in the TEM had confirmed their size, as shown in electronic supplementary material, figure S1. The results confirm that particle size and density does not influence the diffusion of the particle in this regime.

## 4. Discussion

For each combination of particle size, density, viscosity and concentration, at least six particles were tracked independently so that the data presented in figures 1–4 are average values with typical standard deviations shown in figure 4. Six particles were tracked, i.e. *n* = 6, in order for the 98% confidence intervals to be equal to the standard deviation, i.e. the ratio of the standard deviation and margin of error is unity [30], and assuming a normal distribution. The random nature of the measured motion was confirmed by examining the distribution of measured displacements, which were all found to be Gaussian as shown for the 20 and 150 nm diameter polystyrene particles in electronic supplementary material, figure S1. In addition, the mean square displacements exhibited a linear trend, as shown in electronic supplementary material, figure S4, providing additional confirmation of Brownian motion.

In predicting the diffusion of the particles using the classical Stokes–Einstein relationship in equation (2.3), the known physical diameter of the particle was employed rather than the unknown hydrodynamic diameter, which accounts for the geometric core dimension of the particle and the Debye layer of attached charged ions. Hence, the estimates of the diameter at which behaviour transitions from fractional to classical Stokes–Einstein behaviour are based on the physical or core particle diameter. However, Henry's function [31] can be used to translate between these two diameters because it implies a constant ratio of core particle diameter to Debye layer thickness for a given ionic strength.

At the transition to fractional Stokes–Einstein behaviour, classical Stokes–Einstein behaviour would be expected to break down if there is constraint from the container walls or from neighbouring particles and when there is convection or thermal non-equilibrium. However, care was taken to ensure that these conditions did not occur by collecting data away from the influence of the container walls, by using very low concentrations of isolated single particles, and by controlling and monitoring a spatially and temporally constant temperature.

Verpillat *et al.* [32] calibrated their dark-field digital holographic microscope using 100 nm gold nanoparticles at a concentration of 5.6 × 10^6^ particles ml^−1^, i.e. similar to the values used in this study, and observed classical Stokes–Einstein behaviour with a diffusion coefficient of approximately 42 × 10^−13^ m^2^ s^−1^. However, although they captured data for 10 s, they calculated the diffusion coefficient using a linear fit over the first six frames, which Michalet [27] has suggested may lead to misleading results. Our data give similar results when only six frames are used to calculate the diffusion coefficient.

The diffusion coefficient, *D*_T_ for the 150 nm diameter gold particles dispersed in water at a concentration of 5 × 10^−3^ mg ml^−1^ or 0.0005% by weight was about 3 × 10^−13^ m^2^ s^−1^ and an order of magnitude less than *D*_T_ ≈ 30 × 10^−13^ m^2^ s^−1^, which was obtained by Koenig *et al.* [14] for the same type of particle at the as-supplied concentration of 1.66 × 10^9^ particles ml^−1^. However, the as-supplied concentration is ten times more crowded than the highest concentration at which it was possible to track individual particles in this study using the caustic method. He *et al.* [33], Vareene *et al.* [34] and Gollwitzer *et al.* [35] using DLS and Chon *et al.* [36] using fluorescence lifetime correlation spectroscopy (FLCS) observed classical Stokes–Einstein behaviour of particles of the order of 100 nm diameter at concentrations that were at least two orders of magnitude greater than in the experiments reported here. These comparisons support the conclusion from figure 3 that the transition occurs at a smaller diameter of particle as the concentration is increased. Or, to reverse the perspective, at the lowest concentration investigated in this study (5 × 10^−6^ mg ml^−1^), fractional Stokes–Einstein behaviour can be sustained for large particles because there are about 58 billion water molecules for each 100 nm diameter particle. This results in each particle being mostly surrounded by fluid molecules so that its behaviour follows the regime of the fluid; and as the concentration of particles is decreased, it becomes easier to sustain this behaviour for larger particles, hence the transition occurs at larger diameters for lower concentrations. This description provides a sense of scale without dealing with issues associated with the surface energy of the particle; however, there is insufficient evidence to extend it further.

Zwanig & Harrison [37] criticized the use of the fractional Stokes–Einstein equation for molecular fluids on the grounds that molecules cannot be considered hard spheres. However, this criticism is not applicable to our spherical particles, whose shape is confirmed in TEM images from the manufacturer's datasheet and our own observations (electronic supplementary material, figure S4). In addition, in our experimental conditions, the de Broglie wavelengths of the particles are small compared to their diameters and therefore their wave-like behaviour can be ignored.

The experiments reported here were performed at close to ambient conditions; however, the self-diffusion of water has been shown previously [1,2] to follow the fractional Stokes–Einstein relation over a temperature range from 238 to 633 K and so similar behaviour is likely to hold over this temperature range but with different values for the diameters at which the transition behaviour occurs.

## 5. Conclusion

Computational studies have shown that a transition between fractional and classical Stokes–Einstein behaviour exists, but limits on computational power have hindered its evaluation. Our experiments provide the first experimental demonstration of this phenomenon, and in addition show the critical size for transition is dependent on viscosity and concentration. We found the size at which the transition occurs is an inverse function of viscosity and in the range 200–300 nm for the conditions in our experiments.

## Supplementary Material

Transition from fractional to classical Stokes-Einstein behaviour in simple fluids – ESM

## Data accessibility

Our data are deposited at Dryad (http://dx.doi.org/10.5061/dryad.db56v) [38]. See supplementary material for additional information on materials and experimental methods.
