# Supplementary material for: Transition from fractional to classical Stokes–Einstein behaviour in simple fluids
Source: R Soc Open Sci. 2017 Dec 13;4(12):170507. doi: 10.1098/rsos.170507 (PMC5749985; doi:10.1098/rsos.170507)
Supplement: Transition from fractional to classical Stokes-Einstein behaviour in simple fluids – ESM [file rsos170507supp1.docx]

**Transition from fractional to classical Stokes-Einstein behaviour in simple fluids – Supplementary information**


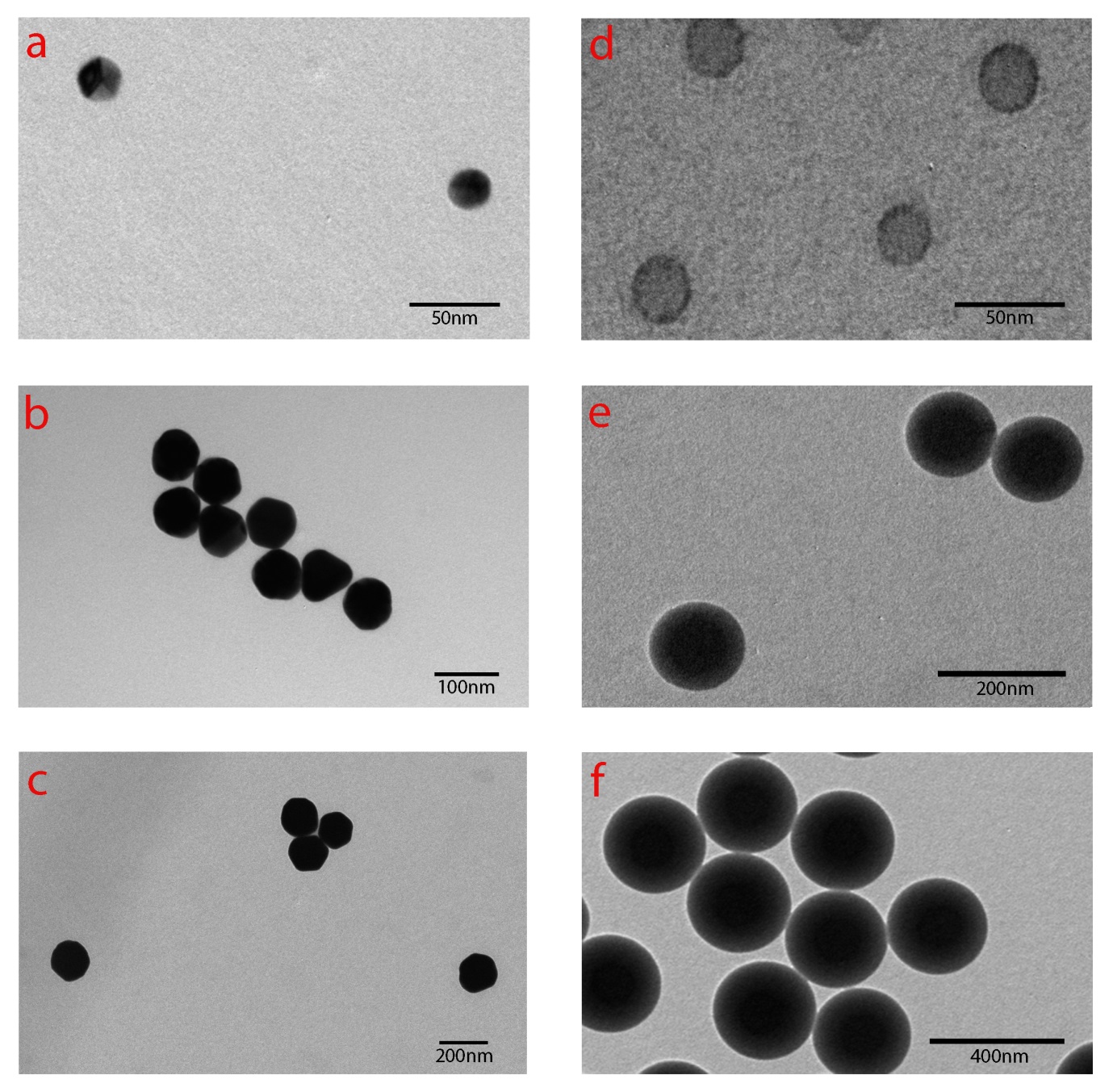


Figure S1 – TEM images confirm nanoparticles spherical shape and size. Solutions of gold nanoparticles at as-supplied concentrations were incubated and then air-dried onto copper grids for 5 minutes. Polystyrene particles were in a similar manner and then stained with aqueous 3% Uranyl Acetate for 30 seconds before drying. 20, 80 and 150nm gold particles (a, b and c respectively) and 20, 150 and 300nm polystyrene particles (d, e and f respectively) were then observed in a transmission electron microscope (Tecnai G2, Fei) at 120kV. The average size of the gold nanoparticles observed in the TEM (calculated over 20 particles for each sample) was respectively 20.69±0.85nm, 79.34±2.91nm and 153.94±8.72nm. For the polystyrene nanoparticles, the average size was found to be, respectively, 20.12±0.92, 156.88±11.32 and 302.23±10.18 nm.


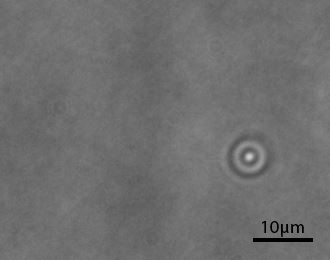


**Figure S2** – Caustic image of a single 10nm gold nanoparticle dispersed in water.


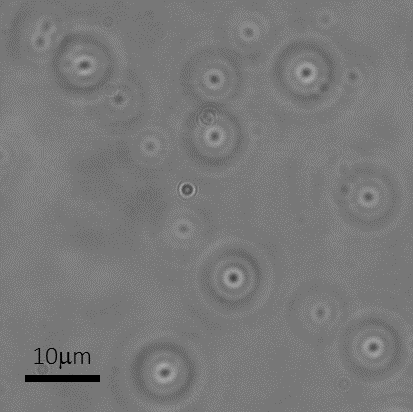


**Figure S3** – Caustic image of a nanoparticle aggregation forming in a high concentration population of 100nm diameter particles showingcaustics of individual particles which are beginning to overlap, from earlier work [15]. In the current work only caustics which were clearly identifiable as a single, isolated pattern were tracked.


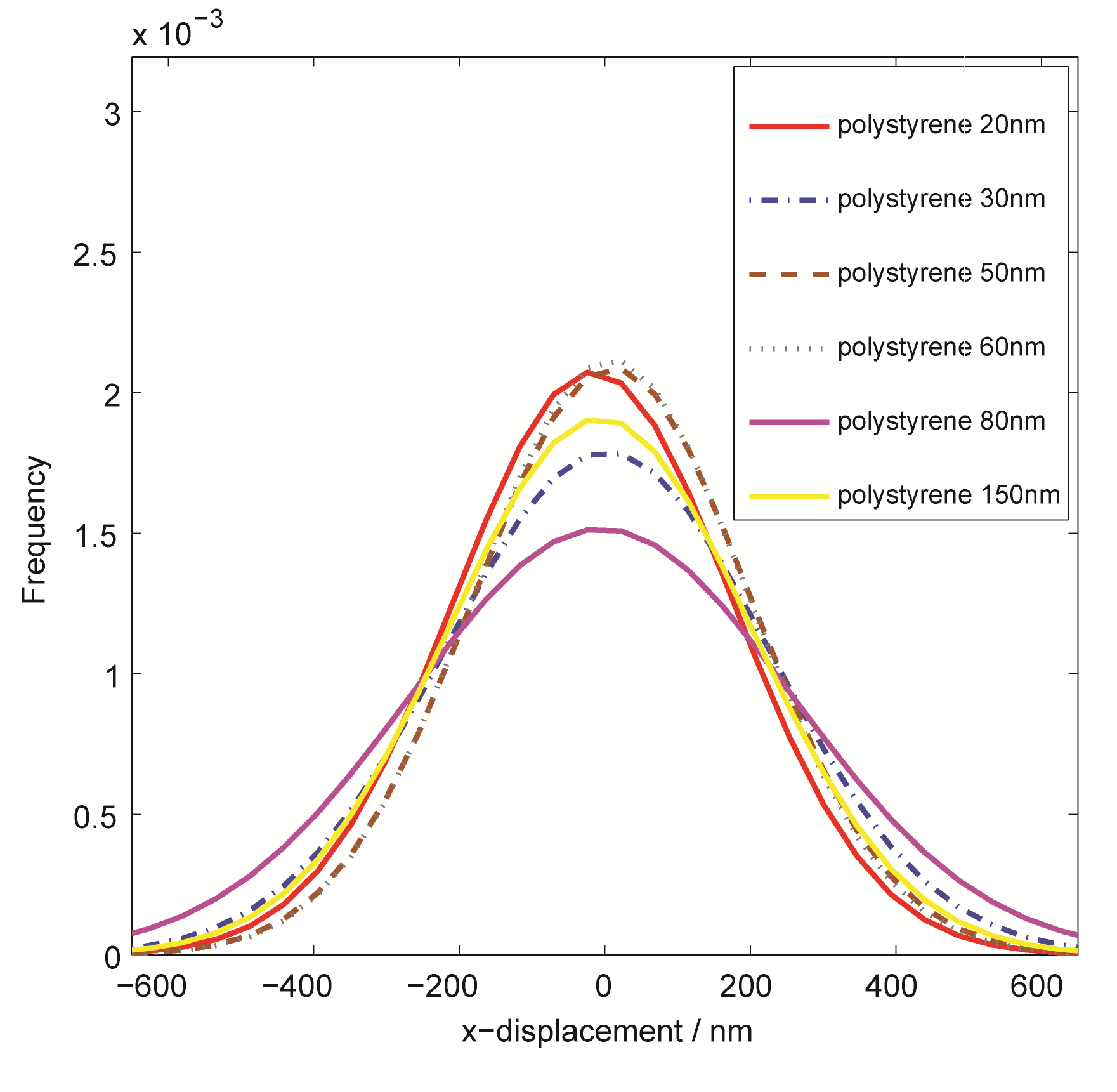


**Figure S4** - Gaussian distributions for a range of polystyrene nanoparticles dispersed in water at 5x10^-6^mg/mL. The displacements data were found, in every case under investigation, to be well-represented by a Gaussian fit. The change in dimension, material, concentration or viscosity did not affect the random nature of the particle motion.


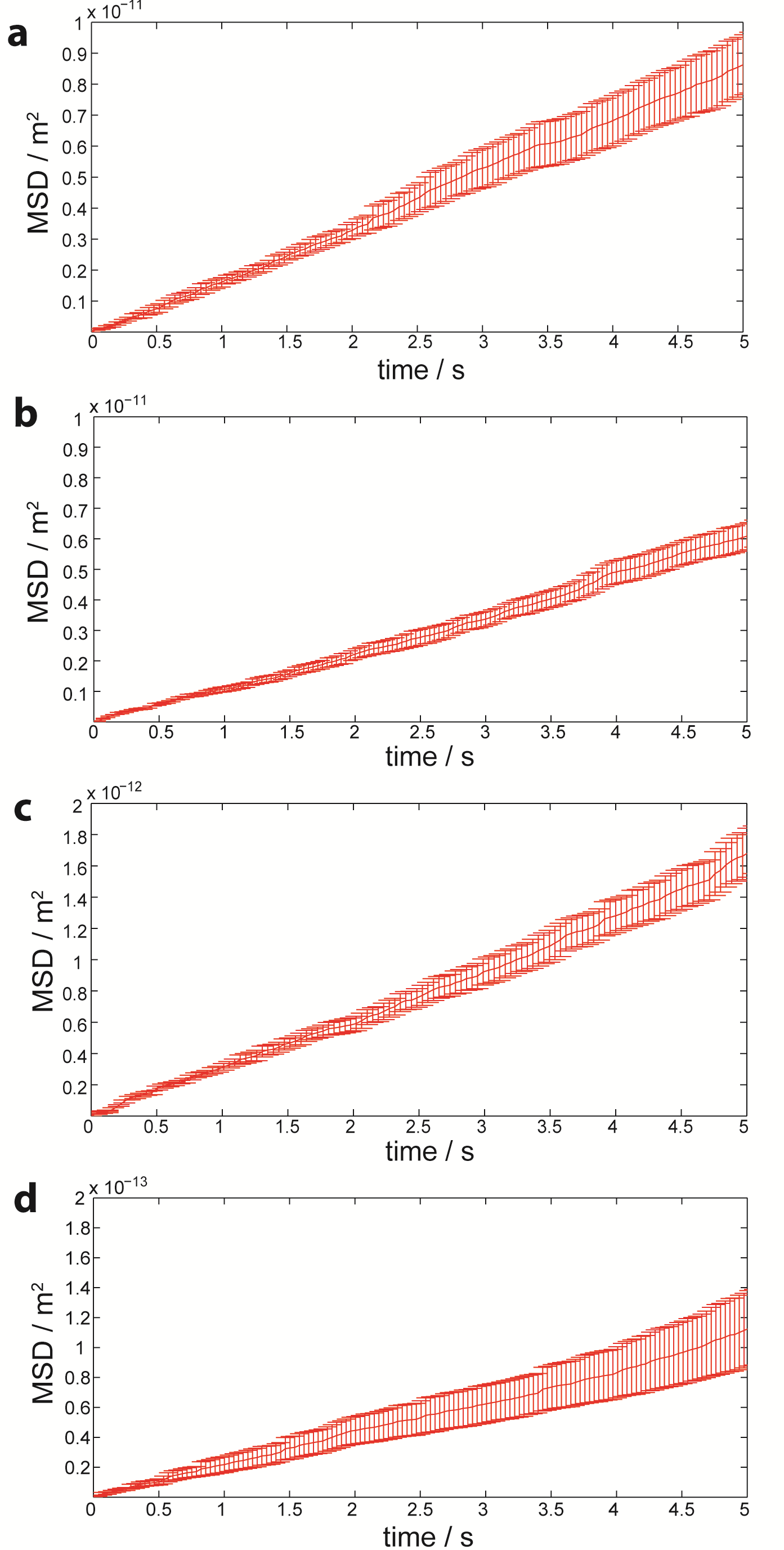


**Figure S5** –Mean square displacement (MSD) of a 50nm gold particle at a concentration of 5x10^-6^mg/ml in: a) water; b) a mixture of 20% of glycerol; c) a mixture of 50% glycerol; and d) a mixture of 90% glycerol. To appreciate the linear trend in c) and d), a change of scale on the y-axis, representing the mean square displacement, was necessary compared to a) and b). The increase in viscosity, due to a higher percentage of glycerol, caused a further decrease in diffusion, as can be noticed by the order of magnitude of the mean square displacement on the y-axis.
